# Preservation of Beef Quality and Myofibrillar Protein Structural and Functional Integrity by Ultrasound-Assisted Immersion Freezing

**DOI:** 10.3390/foods15142412

**Published:** 2026-07-08

**Authors:** Shuo Ye, Chenhao Sun, Bo Chen, Ruihao Niu, Yu Wang, Wuchao Ma, Jiansheng Zhao, Wanli Zhang, Jing Zhao, Junguang Li

**Affiliations:** 1 Food Laboratory of Zhongyuan, Zhengzhou University of Light Industry, Luohe 462300, China; aniye_07@163.com (S.Y.); sunchenhao1010@163.com (C.S.); chenboemail163@163.com (B.C.); niuruihao123@163.com (R.N.); wy92@zzuli.edu.cn (Y.W.); mawuchao66@163.com (W.M.); wlzhang916@163.com (W.Z.); 2 College of Food and Bioengineering, Zhengzhou University of Light Industry, Zhengzhou 450001, China; 3 Henan Shuanghui Investment & Development Co., Ltd., Luohe 462000, China; zjs4567@163.com; 4 Henan Hengdu Food Co., Ltd., Zhumadian 463000, China; wszj1126@126.com

**Keywords:** beef, myofibrillar protein, physicochemical properties, oxidative properties, ultrasound-assisted immersion freezing

## Abstract

This study compared the effects of air freezing (AF), immersion freezing (IF), and ultrasound-assisted immersion freezing (UIF) at different power levels on the physicochemical properties, oxidative characteristics, and the structural and functional properties of myofibrillar protein (MP) of fresh beef. The results showed that UIF treatments generally outperformed AF and IF, with UIF-400 W exhibiting the best performance in maintaining beef quality. Specifically, UIF-400 W mitigated muscle fiber damage by inhibiting ice crystal growth, significantly improved water-holding capacity and color stability, reduced shear force, and effectively delayed protein and lipid oxidation. In addition, UIF-400 W brought the rheological properties, emulsifying properties, water distribution, and gel microstructure of MP closer to those of fresh beef. These findings highlight that UIF-400 W can effectively preserve beef quality as well as the structural and functional characteristics of MP, reduce oxidative damage, and thus become a promising technology for quality improvement of beef during freezing.

## 1. Introduction

Beef is widely consumed due to its high-quality protein and essential nutrients, yet its post-slaughter shelf life is severely limited by rapid biochemical deterioration and microbial spoilage. Freezing is a primary method for prolonging shelf life as it suppresses microbial proliferation and retards unwanted biochemical processes [1]. However, conventional freezing methods such as air freezing (AF) produce large, unevenly distributed ice crystals due to slow freezing rates, which disrupt muscle tissue structure and promote protein and lipid oxidation, leading to significant quality deterioration [2]. Studies by Kowalczyk et al. [3] revealed that freezing alters the physicochemical properties of veal compared with fresh chilled meat, and Al-Dalali et al. [4] also demonstrated that freezing generates ice crystals inside beef, triggering damage to cellular structures and muscle fibers while impairing the chemical and sensory attributes of beef simultaneously.

In recent years, multiple novel assisted freezing techniques such as ultrasound-assisted freezing, magnetic field-assisted freezing and electric field-assisted freezing have been proposed, and their beneficial impacts on meat preservation have been well validated. Among them, ultrasound-assisted immersion freezing (UIF) has attracted particular attention. UIF combines the ice crystal size-reducing effect of ultrasound cavitation with the rapid cooling advantage of immersion freezing (IF). Zhang et al. [5] reported that UIF effectively delayed protein oxidation and loss of thermal stability in chicken breast. Xu et al. [6] demonstrated that multi-frequency UIF reduced ice crystal size in giant river prawn by more than 40%, with less microstructural deformation and reduced protein oxidation. The study by Li et al. [7] demonstrated that UIF could be used to control the quality deterioration of *Pseudosciaena crocea* during repeated freeze–thaw cycles, such as by maintaining muscle integrity and delaying lipid oxidation. Our team has also confirmed the effectiveness of UIF in improving meat quality: it reduced drip loss, enhanced tenderness and color stability in pork [8], and delayed protein structural degradation and thermal instability while improving digestibility [9]. Collectively, these studies suggest that UIF holds significant promise for meat quality preservation.

Despite these promising findings, existing UIF research remains limited in several critical aspects. First, most studies have focused on a narrow range of quality indicators, primarily at the macroscopic level, without systematically examining the relationship between ultrasonic power and comprehensive quality attributes. Second, the effects of UIF on fresh beef, particularly its influence on protein and lipid oxidation pathways, have not been systematically investigated. Third, the impact of UIF on the structural and functional properties of myofibrillar protein (MP), the core structural protein determining beef texture and functionality, remains poorly understood. Macroscopic quality directly determines consumer acceptance, protein and lipid oxidation affects shelf life and safety, and MP structure and functionality form the foundation of meat quality. These unresolved scientific questions hinder the rational optimization and industrial application of UIF technology. Therefore, this study aims to bridge these knowledge gaps through systematic investigation.

In summary, this study used fresh beef as a control to systematically evaluate the effects of AF, IF, and UIF at different power levels (200, 400, and 600 W) on beef quality. The parameters assessed included water-holding capacity (WHC), tenderness, color stability, volatile flavor compounds, microstructure, and oxidative properties, including total sulfhydryl levels, carbonyl content, Ca^2+^-ATPase activity, and thiobarbituric acid-reactive substances (TBARS). In addition, the dynamic rheological behavior, emulsifying activity index (EAI), emulsion stability index (ESI), gel water distribution, and gel microstructure of MP were analyzed. These investigations aimed to elucidate the regulatory mechanism of UIF, identify its optimal parameters, and provide theoretical support for mitigating oxidative damage and maintaining beef quality during frozen storage. Ultimately, this study seeks to promote the application of ultrasound technology in the meat freezing industry.

## 2. Materials and Methods

### 2.1. Materials

For each experimental replicate, beef samples were obtained from the same batch of Xia-nan bull cattle at 24–36 months of age from Henan Hengdu Food Co., Ltd. (Zhumadian, China). Within 24 h postmortem, 1.5 kg of fresh upper hind leg muscle (*gluteobiceps*) was collected from the same carcass and transported to the laboratory at 0–4 °C. All chemicals used in this study were of analytical grade.

### 2.2. Sample Preparation

**Figure 1:**
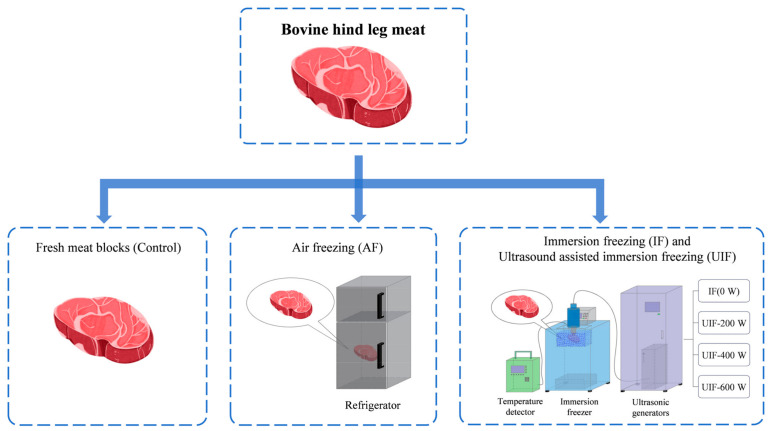
Schematic diagram of different ways of handling beef.

All samples for each experiment were obtained from the same muscle. Once the beef specimens reached the lab, they were immediately divided and processed to remove visible non-muscular tissue on the surface, and subsequently cut into blocks of 8 cm × 8 cm × 4 cm (with the long axis parallel to the muscle fiber direction), each weighing about 95 ± 5 g. Then, the beef was immediately placed in polyethylene zipper bags (14 cm × 20 cm, Thickness 0.08 µm, Zhejiang Mingke Plastics Co., Ltd., Ningbo, China) and placed in an environment at 4 ± 0.5 °C for 4 h. As shown in Figure 1, fresh beef blocks served as the control, while the freezing group was treated with AF, IF and UIF at different powers (ultrasound powers: 200 W, 400 W, 600 W). AF was conducted in a refrigerator at −20.0 ± 0.5 °C, UIF was completed using an UIF device (SJT-2-10 L, Shangjia Biotechnology, Wuxi, China). The instrument ran at a frequency of 20 kHz and featured a continuously adjustable power range from 50–2000 W. During UIF, the samples were fully submerged in the freezing solution (95% ethanol) in the freezing bath, with the temperature set to −20 ± 0.5 °C. The ultrasound device was toggled between active and inactive cycles, each lasting 5 s. Upon the central temperature of beef hitting 0 °C, ultrasonic application was initiated and continued until the temperature dropped to −18 °C, then freezing was stopped. IF was frozen using the same equipment and temperature as UIF, but without activating the ultrasound. Afterward, the beef was frozen for 48 h to stabilize prior to testing. During the freezing process, a multi-channel temperature detector (TP700, Toprie, Shenzhen, China) was used to monitor and record temperature changes. The approximate freezing times (from 4 °C to −18 °C) for each treatment group were as follows: AF 178 min, IF 53 min, UIF-200 W 45 min, UIF-400 W 40 min, and UIF-600 W 43 min.

### 2.3. Thawing Loss and Centrifugal Loss

Based on the methodology established by Gan et al. [10], the weight of beef prior to thawing was designated as *M*_0_ (g). The beef was thawed at 4 °C for 12 h, and after absorbing the exuded water, it was weighed immediately and recorded as *M*_1_ (g). This can be denoted as:

(1) $$Thawing\;Loss\;(\%)=\frac{M_{0}-M_{1}}{M_{0}}\times 100\%$$

Based on the approach mentioned by Wang et al. [11], 8.0 g of beef was weighed out and recorded as *m*_0_ (g), then placed into a tube with a piece of absorbent cotton of equal size at the bottom. The tube was subsequently centrifuged at 10,000× *g* for 10 min in a centrifuge (Avanti J-26s XPI, Beckman Coulter, Brea, CA, USA) at 4 °C, and the resulting mass was recorded as *m*_1_ (g).

(2) $$Centrifugal\;Loss\;(\%)=\frac{m_{0}-m_{1}}{m_{0}}\times 100\%$$

Three parallel replicates were prepared for each treatment group, and the entire experiment was conducted in triplicate.

### 2.4. Tenderness

Based on the approach mentioned by Xu et al. [12], intact raw beef samples were placed in cooking bags (12 cm × 30 cm, Shijiazhuang Xilong Packaging Co., Ltd., Shijiazhuang, China) and heated in a water bath at 80 °C. A multi-channel temperature detector (see Section 2.2 for details) was used to monitor the core temperature of the samples until it reached 75 °C, at which point the samples were removed. Subsequently, the exudates on the beef surface were wiped off, and the beef was stabilized at 4 °C for 4 h. After stabilization, the beef was cut into pieces of 1 cm × 2 cm × 1 cm in size. The texture analyzer (TPA. XT. plus, Stable Micro Systems, Godalming, UK) was employed to determine the shear force of the beef with the HBS probe and the following settings: pre-test speed and test speed were both set at 1 mm/s, and post-test speed was set at 10 mm/s. Each beef sample was tested in triplicate for consistency.

### 2.5. Myoglobin Stability

Following the method of Krzywicki [13], fresh and thawed beef were minced using a meat grinder (FB10E, Supor, Hangzhou, China), and 5 g of minced beef was mixed with 25 mL of phosphate-buffered saline (PBS; 0.04 M, pH 6.8), after which this mixture was homogenized at 10,800 rpm for 1 min at 4 °C with intermittent operation (20 s on and 20 s off). post-homogenization, the mixture was incubated at 4 °C for 1 h to facilitate complete extraction of target components, then subjected to centrifugation at 10,000× *g* (4 °C) for 20 min, and the resulting supernatant was harvested for downstream analyses, whose absorbance (*A*) was assayed at the 525, 545, 565, and 572 nm with a spectrophotometer (TU-1810, Persee, Beijing, China). Relative concentrations of oxymyoglobin (OxyMb) and metmyoglobin (MetMb) in the samples were computed using the formulas listed below:

OxyMb (%) = (0.882 *R*_1_ − 1.267 *R*_2_ + 0.809 *R*_3_ − 0.361) × 100 (3)

MetMb (%) = (−2.514 *R*_1_ + 0.777 *R*_2_ + 0.800 *R*_3_ + 1.098) × 100 (4)

Here, *R*_1_ = *A*_572_/*A*_525_, *R*_2_ = *A*_565_/*A*_525_, *R*_3_ = *A*_545_/*A*_525_. The experiment was carried out in three independent replicates, each containing three parallel samples per treatment group.

### 2.6. Electronic Nose

Based on the approach mentioned by Chen et al. [14], an electronic nose (PEN3 electronic nose, Airsense, Schwerin, Germany) was utilized to conduct volatile flavor analysis of beef samples to differentiate aroma compounds. Approximately 3 g of minced raw beef samples from each group were weighed and sealed in specific headspace vials for the electronic nose. These samples were subsequently exposed to a 45 °C water bath for 30 min, then the gases inside the vials was analyzed. The detection parameters are as follows: carrier gas: air; temperature: 25 °C; preparation time: 5 s; purge time: 120 s; injection flow rate was 60 mL/min; testing duration: 250 s. The signals acquired within 230 s were applied to radar image analysis. Additionally, data collected 230–232 s were specifically used for principal component analysis (PCA). Three independent runs of the experiment were conducted, with three replicates per group in each run.

### 2.7. Microstructure

With slight modifications, the method employed in this study was based on that of Wang et al. [15]. The raw beef (4 °C) was cut into 1 cm × 1 cm × 0.5 cm cubes, with cuts made both perpendicular and parallel to the fiber direction. Subsequently, these beef cubes were embedded in OCT compound on a specialized sample holder for a cryostat microtome (CryoStar NX50, Thermo Fisher Scientific, Waltham, MA, USA). After being frozen and solidified in a −60 °C quick-freezing chamber, the beef cubes were sectioned into approximately 10 μm slices at −20 °C, yielding both cross-sectional and longitudinal sections of the beef. Immediately after slicing, the samples were placed on microscope slides. They were washed three times with 75% ethanol for dehydration and then stained with eosin dye for 30 s. After excess dye was absorbed with filter paper, a coverslip was placed over the sections. The slides were then viewed with a polarized light microscope (BX53M, Olympus Corporation, Tokyo, Japan) at 50× magnification to observe the morphology of ice crystals. The experiment was performed in three independent replicates, and one representative result image was selected.

### 2.8. Protein Oxidation

#### 2.8.1. Total Sulfhydryl Content

The method employed for the determination followed that of Kang et al. [16]. Chopped raw beef samples (4 g) were added to 36 mL KCl (0.6 M), and homogenized at 10,000 rpm for three cycles, each lasting 30 s. The filtrate of the homogenized liquid filtered by gauze was taken 0.4 mL and introduced into 10 mL of PBS (0.1 M KH_2_PO_4_/K_2_HPO_4_, 1 mM EDTA, 8 M urea, pH 8.0). DTNB (0.2 mL, 10 mM) was introduced into the blend, thoroughly mixed, and then left protected from light for 15 min. Subsequently, the resultant blend was centrifuged (4500× *g*, 5 min), and absorbance of the centrifugal supernatant was assayed at 412 nm. Three independent runs of the experiment were conducted, with three replicates per group in each run.

#### 2.8.2. Carbonyl Content

Based on the approach mentioned by Al-Dalali et al. [4]: For each treatment group, three samples of raw minced beef (3 g each) were prepared. Each 3 g sample was mixed with 30 mL of 20 mM PBS, and the blend was subjected to homogenization for 30 s. Then, 0.5 mL of 10 mM DNPH was introduced and mixed thoroughly; the blend was incubated protected from light at 37 °C for 1 h before 0.5 mL of 20% TCA was introduced. Then, the resulting homogenate was centrifuged at 17,000× *g* for 15 min at 4 °C; the protein precipitate was washed and centrifuged three times with 1 mL of 1:1 (*v*/*v*) ethanol–ethyl acetate mixture under the same parameters. Subsequently, the pellet was redissolved in 1 mL of 6 M guanidine hydrochloride and placed in a 37 °C water bath until it dissolved completely. Then, the system was centrifuged again at 17,000× *g* for 10 min; carbonyl content was determined via the absorbance of the upper fraction at 370 nm. The experiment was performed in 3 independent replicates.

#### 2.8.3. Ca^2+^-ATPase Activity

The Ca^2+^-ATPase kit (BC0965, Soleibao, Beijing, China) served to determine the activity of Ca^2+^-ATPase. For each treatment group, three samples of raw minced beef (1 g each) were each mixed with 10 mL of PBS (20 mM, pH 7.2, incorporating 0.6 M NaCl) and homogenized at 8000 rpm for 30 s. After an enzymatic reaction (37 °C, 10 min), add 10% TCA to bring the reaction to a stop. Subsequently, centrifuge the blend at 4000× *g* for 10 min, collect the supernatant, add a phosphorus-fixing agent, and incubate in a 40 °C water bath for 10 min. Once cooled, the absorbance was quantified at 600 nm. The experiment was performed in 3 independent replicates.

### 2.9. Lipid Oxidation

The method employed for the determination follows that described by Zhang et al. [17]. For each treatment group, three samples of minced raw beef (5 g each) were prepared. Each sample was mixed thoroughly with 25 mL of TCA (17.5% *w*/*v*; with 0.1% EDTA). Homogenize the mixture for 30 s, repeating the process three times, followed by filtration. Add 5 mL of the filtrate to an equivalent volume of 0.02 M TBARS solution, and incubate the blend in a boiling-water bath for 30 min. After allowing the blend to cool gradually for 1 h, add 5 mL of chloroform. Once cooled, the blend was shaken vigorously and left undisturbed for 15 min to facilitate phase separation, the upper phase was then collected, and the absorbance of this collected phase was gauged at 532 nm and 600 nm. The experiment was independently repeated three times.

### 2.10. Extraction of MP

Based on the approach mentioned by Li et al. [18], the minced raw beef was mixed with a 4-fold volume (*w*/*v*) of PBS (10 mM, with 2 mM MgCl_2_, 0.1 M NaCl, 2 mM EGTA, pH 7.0, 4 °C) and homogenized three times. The homogenate was centrifuged (3000× *g*, 4 °C, 15 min), and the pellet was retained. The above process was reproduced 3 times. Then, the pellet was subjected to three washes with an equal volume of 0.1 M NaCl (4 °C) under the same centrifugation parameters, thus yielding the final precipitate, namely MP. The obtained MP was placed in a beaker, hermetically sealed, and held at 4 °C for utilization within 2 d. MP concentration was assayed using the biuret procedure, and PBS (10 mM, with 0.6 M NaCl, pH 7.0, 4 °C) was used to adjust the protein concentration.

### 2.11. Functional Properties of MP

#### 2.11.1. EAI and ESI

EAI and ESI of beef MP were quantified via the protocol from Zhang et al. [19]. Briefly, the MP was thinned out to a concentration of 4 mg/mL with PBS. It was then amalgamated with the soybean oil phase at a 4:1 volume ratio, and homogenized at 1 min for 60 s via a homogenizer. Immediately after homogenization, 50 μL of the emulsion was collected from the same position at the bottom of the container and added to 4.95 mL of SDS solution (0.1%, *w*/*v*), followed by thorough mixing. The absorbance of this mixture was checked at 500 nm, using the SDS solution by itself as the blank reference. After the emulsion was left undisturbed for 10 min, the absorbance value at the same position was ascertained again using the same method. The calculation protocols for EAI and ESI are as follows.

(5) $$\mathrm{EAI}\;(m^{2}/g)\;=\frac{2\times 2.303\times A_{0}\times N}{\rho \times (1-\varphi )\text{×}10^{4}}$$

(6) $$ESI\;(\%)=\frac{A_{10}}{A_{0}}$$

Here, *A*_0_ and *A*_10_ denote the emulsion’s absorbance readings at 0 min and 10 min, respectively; *N* is the dilution factor (100); *ρ* is the MP concentration (g/mL); and *φ* is the oil volume fraction (20%). Three independent runs of the experiment were conducted, with three replicates per group in each run.

#### 2.11.2. Dynamic Rheological Properties

Following the method of Wang et al. [20], approximately 1 g of the MP solution was situated on the lower plate (40 mm) of a parallel-plate system on a rotational rheometer (DHR-1, TA Instruments, New Castle, DE, USA). With a gap distance set at 1 mm, the MP solution was then heated from 20 °C to 80 °C, and the storage modulus (G′) was recorded throughout the test. The experiment was carried out in 3 independent replicates, each containing 3 parallel samples per treatment group.

#### 2.11.3. Preparation of MP Gel

Based on the approach mentioned by Zhang et al. [21], an MP (40 mg/mL) was prepared using PBS and centrifuged at 500× *g* for 2 min to expel bubbles. The MP solution was then transferred to a beaker, heated in an 80 °C water bath for 0.5 h, taken out and cooled in an ice water bath. Subsequently, the samples were refrigerated for 12 h.

#### 2.11.4. Moisture Distribution

Based on the methodology mentioned by Shi et al. [22], the relaxation time (T_2_) of the MP gel was analyzed using a low-field nuclear magnetic resonance (LF-NMR) analyzer (NMI20-060V-I, Niumag, Suzhou, China). For each treatment group, three 1.5 g MP gel samples were prepared. Each sample was placed into an 8 mm diameter NMR tube. The magnetic field strength was set at 0.5 ± 0.08 T, and the proton resonant frequency was 18 MHz. The transverse relaxation time (T_2_) and corresponding peak area fractions (P_2_) were determined using the CPMG sequence. The experiment was carried out in three independent runs.

### 2.12. Microstructure of MP Gel

#### 2.12.1. Confocal Laser Scanning Microscopy (CLSM)

Based on the approach mentioned by Wang et al. [20], CLSM (FV3000, Olympus, Tokyo, Japan) was applied to characterize the microstructure of protein gels. The MP gels were sectioned into tiny 2 mm × 2 mm × 1 mm cubes and dyed with Rhodamine B (0.1% *w*/*v*, dissolved in 0.1 M PBS, pH 7.2) for 8 min, rinsed 3 times, then placed in a glass-bottomed dish for CLSM observation. The excitation wavelength, emission wavelength range, and objective lens magnification of CLSM were set to 561 nm, 570–620 nm, and 20×, respectively. The experiment was repeated three times, and one image from each group was selected as the result.

#### 2.12.2. Scanning Electron Microscopy (SEM)

The microstructure of the gels was further visualized with a scanning electron microscope (SEM, SU-8010, Hitachi, Tokyo, Japan). Based on the approach proposed by Zhang et al. [19] with slight tweaks, the MP gels were sliced into 3 mm × 3 mm × 2 mm fragments, steeped in glutaraldehyde (2.5% *w*/*v*, pH 7.2), and kept in a 4 °C dark condition for 12 h. Subsequently, the gels were rinsed with 0.1 M PBS (pH 7.2) for 10 min, and this rinsing step was repeated three times. Then, dehydration was performed with 50%, 75%, 90%, and 99.5% ethanol sequentially for 10 min each. After dehydration, the gels were immersed in a mixture of equal volumes of anhydrous ethanol and tert-butanol, followed by pure tert-butanol, for 15 min respectively. Finally, the gels were freeze-dried for 36 h, subjected to gold sputtering, and observed and photographed under SEM at a magnification of 2000×. Representative images from three independent replicates for each group.

### 2.13. Statistical Analysis

All experiments were performed with three independent replicates, each with three parallel subgroups. Data for thawing loss, centrifugal loss, shear force, myoglobin stability, total sulfhydryl content, carbonyl content, Ca^2+^-ATPase activity, TBARS, EAI, ESI, and the proportions of water types in MP gel were analyzed by one-way ANOVA using SPSS Statistics 25. Significant differences were determined by Duncan’s multiple range test at *p* < 0.05, and outcomes are reported as mean ± standard deviation. PCA and data plotting were performed using Origin 2022 (OriginLab Corporation, Northampton, MA, USA).

## 3. Results and Discussion

### 3.1. WHC of Beef

The thawing loss and centrifugation loss of frozen beef can clearly indicate the WHC of the beef sample, which is a key indicator representing the quality of beef products [23]. As shown in Table 1, samples treated with AF exhibited significantly higher thawing loss (6.58%) and centrifugal loss (16.87%) compared to other treatment groups (*p* < 0.05). In contrast, the loss rate of beef processed by UIF was diminished, and the loss rates also dropped with the elevation of ultrasound power. Specifically, beef treated with UIF at 400 W had the lowest thawing loss (2.83%) and centrifugal loss (11.75%). The underlying cause lies in the mechanical actions and cavitation phenomena generated by 400 W ultrasonic treatment, which lead to cell elongation and increased internal space within muscle fibers, thereby enhancing WHC [24]. This also improves the structural characteristics of proteins, reduces their aggregation degree, thereby increasing their WHC and affecting beef quality [11]. However, under UIF treatment at 600 W, the thawing loss (4.21%) and centrifugal loss (16.68%) of the beef increased significantly. The reason is that excessively high ultrasonic power induces severe structural destruction of MP, leading to irreversible water loss in the protein [25]. Additionally, high-intensity ultrasound can excessively damage muscle fibers, causing obvious cracks and loss of water molecules, reducing the WHC of the sample [26,27].

### 3.2. Tenderness of Beef

**Figure 2:**
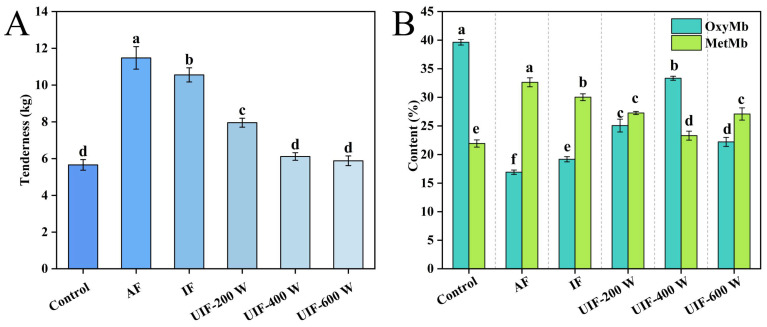
The shear force (**A**) and color stability (**B**) of beef under different freezing treatments. Significant differences among samples (*p* < 0.05) are denoted by different alphabetical notations.

Tenderness acts as a critical index in gauging the quality of meat, directly influencing consumer acceptance. The shear force value represents the resistance of muscle fibers to shear force and directly reflects muscle tenderness. Less pronounced shear force corresponds to a higher level of tenderness in the beef [28]. As shown in Figure 2A, the AF group had the highest shear force (11.81 ± 0.55 kg), which was different from other treatment groups (*p* < 0.05). This originates from the reality that bulk ice crystals generated during the slow freezing process damage the muscle structure; these large ice crystals cause significant water loss during thawing, thereby impacting the tenderness of beef [8]. The shear force of beef treated with UIF decreased as the ultrasound power intensified. Specifically, the shear force of beef treated with UIF-400 W (6.12 ± 0.21 kg) exhibited no significant difference from the control (5.66 ± 0.29 kg) (*p* > 0.05). Although the shear force of the IF (10.56 ± 0.38 kg) and UIF-200 W (7.96 ± 0.24 kg) also decreased, they remained markedly more elevated than the control (*p* < 0.05). When different ultrasonic power levels are applied to beef samples, they alter the spatial structure of proteins to varying degrees and cause damage to muscle fibers, thereby resulting in this phenomenon [29]. The tenderness of beef is tightly linked to WHC, with higher WHC resulting in greater tenderness, a correlation that is consistent with the findings pertaining to WHC, although cooking loss was not examined here. Future research is needed to further clarify its relationship with tenderness. However, excessively high ultrasound power can break down muscle fibers, damaging their integrity and leading to a relaxed muscle structure and reduced shear force [30].

### 3.3. Stability of Beef Myoglobin

Myoglobin is the primary factor influencing beef color. During refrigerated storage, the auto-oxidative modification of myoglobin converts the bright red oxymyoglobin (OxyMb) into the brownish metmyoglobin (MetMb), altering the beef color and affecting its appearance and consumer acceptance [31]. Generally, greater color stability in beef is associated with reduced MetMb accumulation. When the relative content of MetMb exceeds 60%, the color of fresh beef is not acceptable to consumers. The auto-oxidative process of myoglobin also promotes the oxidative modification of fat and protein [32]. As shown in Figure 2B, the content of OxyMb and MetMb showed an opposite trend. The control had the highest relative content of OxyMb and the lowest relative content of MetMb. Due to slow freezing, the AF was exposed to external factors such as microorganisms, oxygen, and temperature for an extended period, which oxidizes OxyMb to produce more MetMb, turning the beef color into the least preferred brownish hue by consumers. This occurs because the divalent iron ions in myoglobin are oxidized to trivalent iron ions during the prolonged freezing and thawing process, converting OxyMb to MetMb and resulting in beef color deterioration [33]. UIF treatment can truncate the freezing time and reduce the impact of prolonged freezing. Appropriate ultrasound can also activate the reductase system of MetMb, reducing its content. However, excessive ultrasound power may disrupt the structure of water molecules, generating reactive oxygen species that promote the oxidation of myoglobin [34]. Among them, the UIF-400 W treatment was optimal, with myoglobin stability closest to the control.

### 3.4. Volatile Flavor Compounds in Beef

**Figure 3:**
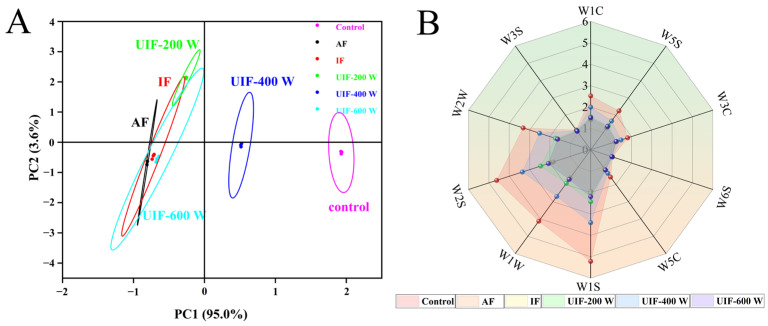
The volatile flavor compounds of beef ((**A**) PCA plot; (**B**) radar plot of electronic nose sensor responses) under different freezing treatments.

The electronic nose is an efficient, rapid, and environmentally friendly technology for distinguishing volatile flavor compounds in samples. By utilizing highly sensitive sensors, it can differentiate and detect subtle changes in aroma components within samples [35]. Figure 3A presents the results of PCA derived from data generated by electronic nose flavor measurements. PCA represents a multivariate statistical analytical method employed for assessing the consistency and variation between substances. Generally speaking, the degree of dispersion of functional groups represented by different treatment groups represents the differences in flavor [36]. The explained variance ratios of the PC1 and the PC2 were 95% and 3.6%, respectively, with a cumulative contribution rate of 98.6% (greater than 80%), which may exhibit the comprehensive flavor characteristics of beef. Beef samples from different treatments showed distinct clustering, with the control and UIF-400 W data concentrated in the first and fourth quadrants and having the closest cluster points. This indicated that the flavor characteristics of beef samples treated with UIF-400 W were closest to those of the control. Data points from other treatment groups were concentrated in the second and third quadrants, far from the control, suggesting significant flavor differences. Some flavor amino acids are lost during the freezing process, which leads to flavor deterioration, thereby resulting in this outcome [37]. Each sensor has different response levels, with sensors W1S, W2S, and W1W showing the strongest responses, corresponding to methyl substances, alcohol substances, and inorganic sulfides, respectively (Figure 3B). Among them, each sensor had a strong response intensity to the control’s beef samples, followed by the UIF-400 W, and then by UIF-200 W, UIF-600 W, IF and AF. This suggested that UIF treatment at 400 W can potently upgrade the flavor features of frozen beef and reduce flavor deterioration.

### 3.5. Microstructure of Beef

**Figure 4:**
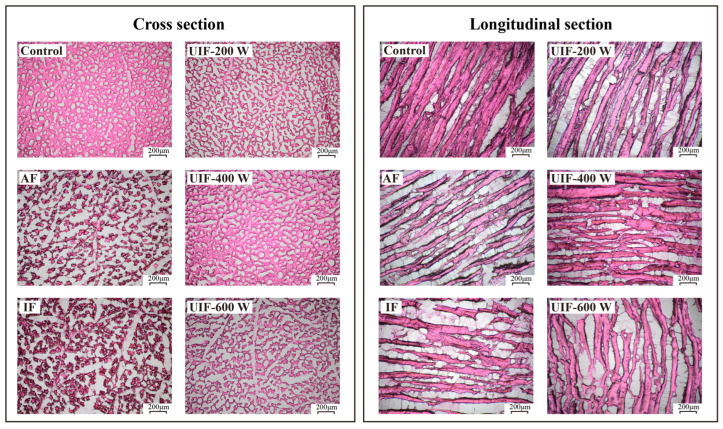
The microstructure of beef under different freezing treatments, magnification: 50×.

During the freezing process of beef, both the growth rate and size of ice crystals affect the changes in muscle structure, altering the arrangement of muscle fibers and subsequently influencing meat quality [38]. Red represents muscle fibers, and white represents the voids created by ice crystals (Figure 4). In the control, the muscle fibers were tightly and neatly arranged. Other samples that underwent freezing treatment showed varying degrees of damage to their muscle fibers. Specifically, the AF exhibited twisted and broken muscle fibers due to the formation of large-volume ice crystals in the non-rapid freezing, with blurred fiber margins and the most extensive white voids, indicating the most severe muscle structure destruction. While incomplete rigor mortis might theoretically contribute to fiber twisting, the extensive white voids and blurred fiber margins observed specifically in the AF group indicate that ice crystal growth during slow freezing was the primary cause, as all samples shared the same pre-freezing rigor status. However, observation from both cross-sectional and longitudinal sections revealed that UIF-400 W exhibited the most intact and densely arranged muscle fiber architecture among all freezing treatments, along with a relatively smaller proportion of white void areas. The underlying mechanism lies in the ability of appropriate ultrasound treatment to break large ice crystals into small and dense ones, which do not cause severe damage to muscle fibers [39]. Sun et al. [40] likewise observed that the collapse of cavitation bubbles and the microjet effect generated via proper ultrasonic processing can alleviate harm to the muscular framework of carp.

### 3.6. Protein Oxidation of Beef

#### 3.6.1. Total Sulfhydryl Content of Beef

Total sulfhydryl content in proteins serve as key metrics for assessing protein oxidation processes in beef items. During frozen storage, protein sulfhydryl groups are susceptible to oxidation, forming disulfide bonds and various sulfur oxides. The generation of disulfide bonds may trigger protein crosslinking and denaturation, bringing about a decline in sulfhydryl content and compromised beef quality [41]. Table 2 shows that compared with the control, the total sulfhydryl content of beef treated with AF significantly decreased (43.41 ± 0.25 nmol/mg·prot). This was accountable to the reality that AF required a long freezing time to fully freeze the beef blocks, which made the beef more prone to oxidative deterioration. With the intervention of ultrasound, the magnitude of the reduction in total sulfhydryl content in the UIF groups decreased. The total sulfhydryl content of IF, UIF-200 W, and UIF-400 W decreased to 43.76 ± 0.41 nmol/mg, 50.28 ± 0.31 nmol/mg, and 56.38 ± 0.09 nmol/mg, respectively. This trend was attributed to the fact that ultrasound accelerated the freezing rate, allowing beef to be frozen more rapidly. In addition, the cavitation effect of ultrasound reduced ice crystal size and alleviated damage to protein structure caused by ice crystals, thereby decreasing the degree of protein oxidation. However, when treated with UIF-600 W, the total sulfhydryl content of the beef decreased to 48.38 ± 0.67 nmol/mg. Excessively high ultrasonic power caused denaturation of protein structure, exposed the intrinsic sulfhydryl groups of proteins, and thereby rendered them more susceptible to oxidation by oxidizing factors. Gülseren et al. [42] also found that ultrasound could reduce the sulfhydryl content in bovine serum albumin.

#### 3.6.2. Carbonyl Content of Beef

Protein carbonylation is an irreversible oxidative modification that occurs on the side chains of amino acids such as lysine and arginine [43], which permanently impairs the structure and function of proteins and directly compromises beef quality. As shown in Table 2, UIF effectively mitigated this carbonylation: the carbonyl content of beef in the AF group was the highest at 1.77 ± 0.03 nmol/mg·prot, which was attributable to the extremely slow freezing rate of air freezing, where ice crystals grew continuously during the prolonged freezing process, severely disrupting the structure of myofibrillar proteins and exposing more amino acid side chains to reactive oxygen species and pro-oxidants, thereby accelerating the carbonylation reaction [44]. All UIF-treated groups showed significant reductions (*p* < 0.05), among which UIF-400 W exhibited the most pronounced decrease, with the carbonyl content falling to 1.16 ± 0.03 nmol/mg·prot. This value was statistically comparable to the control group (1.15 ± 0.03 nmol/mg·prot, *p* > 0.05), indicating that UIF-400 W preserved the resistance of proteins to carbonylation to a level comparable to that of fresh beef. The favorable results of UIF-400 W can be attributed to two key effects: on the one hand, the shorter freezing time of UIF shortens the window of protein exposure to oxidative stress, reducing the chance of amino acid side chains being attacked by free radicals; on the other hand, the ultrasonic power of 400 W achieves an optimal balance between mechanical disruption and cavitation effects, which gently inhibits the development of oversized ice crystals without causing excessive protein denaturation. This balance maximally suppresses the oxidation of amino acid side chains.

In contrast, the carbonyl content of the UIF-600 W group was significantly higher than that of the UIF-400 W group (*p* < 0.05), which was likely because excessively high ultrasonic power generated intense cavitation effects and mechanical stress, directly destroying the secondary and tertiary structure of proteins and exposing more buried amino acid side chains to the oxidative environment. Meanwhile, excessive cavitation might also promote the release of pro-oxidative enzymes and free iron from organelles, forming a pro-oxidative microenvironment that offsets the protective effects of rapid freezing and leads to a rebound in the degree of carbonylation.

#### 3.6.3. Ca^2+^-ATPase Activity of Beef

ATPase activity serves as a well-recognized indicator for evaluating protein structural integrity, with Ca^2+^-ATPase activity specifically employed to define the intactness of myosin. Reports indicate that a decrease in Ca^2+^-ATPase activity may be related to myosin hydrolysis, and the oxidation of sulfhydryl groups at the active sites of myosin can also lead to a reduction in Ca^2+^-ATPase activity [45]. As presented in Table 2, the Ca^2+^-ATPase activities of the groups (listed from top to bottom) were 33.46 ± 1.07 U/g, 21.68 ± 1.09 U/g, 23.32 ± 0.66 U/g, 26.66 ± 0.23 U/g, 32.35 ± 0.60 U/g, and 25.61 ± 0.54 U/g, respectively. This demonstrated that UIF treatment preserves the structural integrity of beef proteins and maintains Ca^2+^-ATPase activity to a certain extent. One plausible explanation for this is that UIF reduced the freezing duration of beef and delayed the reorganization of protein molecules as well as the oxidative modification of sulfhydryl groups within the active domains of myosin molecules. Additionally, this treatment mitigated mechanical damage to muscle fibers, which reduced the denaturation along with inactivation of enzymes with antioxidant properties, thus preserving the functional activity of Ca^2+^-ATPase [46]. This corresponded to the data on total sulfhydryl content.

### 3.7. Lipid Oxidation of Beef

**Figure 5:**
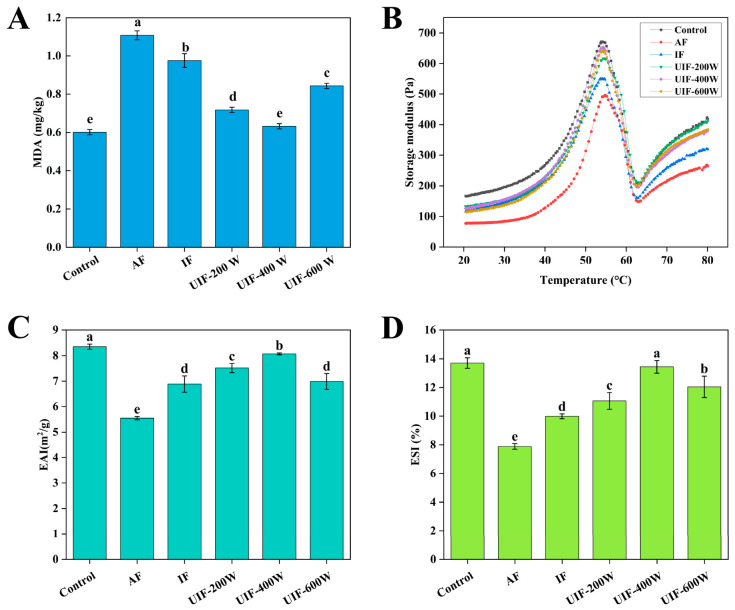
The impacts exerted by diverse freezing regimens on lipid oxidation of beef (**A**), storage modulus (G′) of MP solution during heating (**B**), and EAI (**C**), and ESI (**D**) of MP emulsion. Significant differences among samples (*p* < 0.05) are denoted by different alphabetical notations.

Lipids are widely present in beef as well as its processed products. Lipid oxidation produces a large number of intermediate products, which can also participate in protein oxidation reactions, exacerbating the degree of protein oxidation. After the primary oxidation products of lipids, such as hydroperoxides, are formed, they undergo further oxidative degradation into secondary oxidation products such as ketones, alcohols, and aldehydes. Among these, malondialdehyde (MDA) is the primary substance that can react with thiobarbituric acid (TBA) to produce a red-brown compound (TBARS), so MDA content typically serves as an indicator of lipid oxidation levels [47]. Figure 5A demonstrates that with increasing ultrasound power during UIF treatment, the MDA content first decreased and then increased, and it remained lower than the MDA content of the AF treatment group (1.11 ± 0.02 mg/kg). The MDA content of the UIF-400 W (0.63 ± 0.01 mg/kg) most resembled the control (0.60 ± 0.01 mg/kg), while the MDA content of the UIF-600 W (0.84 ± 0.01 mg/kg) showed an increasing trend. The cavitation effect induced by high-intensity ultrasound power triggers the production of reactive oxygen species, boosting lipid-protein oxidation levels. Moderate lipid oxidation contributes to desirable beef flavor, yet overoxidation simultaneously deteriorates flavor quality and expedites spoilage of beef. UIF treatment can slow down this lipid oxidation. Zou et al. [48] found in a study that ultrasound during cooking increases lipid oxidation in beef.

### 3.8. Functional Properties of MP

#### 3.8.1. Dynamic Rheological Properties of MP

The viscoelasticity of MP is commonly evaluated through dynamic rheological analysis, where the G′ is used to reflect elastic changes within the gel network [49]. Figure 5B depicts G′ profiles throughout the heating process of MP solutions in different freezing groups. G′ magnitudes maintained a fairly steady state over the range of 20 °C to 40 °C, then underwent a sharp elevation near the 40 °C threshold, attaining a maximum value at around 54 °C. This behavior is attributed to the clumping and structural alteration of myosin heads (heavy meromyosin) and the initial establishment of a protein gel structure [50]. Subsequently, G′ decreased sharply, reaching a trough around 63 °C, before steadily increasing again up to 80 °C. The initial decline is due to the conformational change of light meromyosin [51]. The subsequent increase stems from irreversible interconnection between myosin fibrils, a process driven by covalent disulfide linkages alongside hydrophobic associations, which in turn brings about the development of a stable 3D gel structure [52].

MP from beef subjected to different freezing methods exhibited lower G′ values than from fresh beef. The AF exhibited the minimum G′ throughout the heating process, which can be attributed to protein structural damage caused by freezing, bringing about changes to the native conformation and structural unfolding of the proteins. Greater protein denaturation resulted in lower G′ values [53]. In contrast, the UIF groups showed significantly higher G′ than the AF, with G′ basically increasing as ultrasonic power increased. This indicated that ultrasound treatment positively improved the viscoelastic properties and gel network formation of MP during freezing [54]. These results aligned with observations from Li et al. [18], which similarly noted that high-power ultrasound augmented the G′ of chicken MP.

#### 3.8.2. EAI and ESI of MP

The EAI and ESI values for each treatment group are shown in Figure 5C,D, from which it can be seen that UIF significantly alleviated the adverse effects of freezing on the EAI and ESI of MP emulsions. Compared with the control, all freezing treatments resulted in decreased EAI and ESI values, with the AF showing the most pronounced reductions—by 33.6% and 42.52%, respectively (*p* < 0.05). This can primarily result from protein denaturation alongside the development of insoluble aggregates induced by freezing, which hinder the attachment of MP to the surface of oil droplets and consequently impair emulsification properties.

By comparison, the UIF groups exhibited differential levels of enhancement in both EAI and ESI relative to the AF group. These parameters ascended first and then diminished with the escalation of ultrasonic power, attaining the maximum value at 400 W, where EAI and ESI were significantly enhanced to 8.07 m^2^/g and 13.44%, respectively (*p* < 0.05). This trend closely aligned with the observations reported by Liu et al. [55]. The improvement could stem from the cavitation phenomena induced by UIF, which produce microjets and shear forces that disrupt freezing-induced protein aggregates, reduce the negative impact of large aggregates on oil–water interface adsorption, and expose more hydrophobic groups. These changes enhance hydrophobic associations between protein molecules and oil droplets, promoting the attachment process and improving EAI. Meanwhile, turbulence and shear forces promote moderate protein unfolding, modulate charge distribution, enhance electrostatic repulsion, help maintain the integrity of the interfacial protein layer, and resist droplet coalescence, thereby increasing the ESI of MP emulsions [56]. A reduction in EAI and ESI observed at 600 W may result from excessive ultrasound power inducing protein aggregation, leading to reduced adsorption rate and surface hydrophobicity [57].

#### 3.8.3. Moisture Distribution of MP Gel

**Figure 6:**
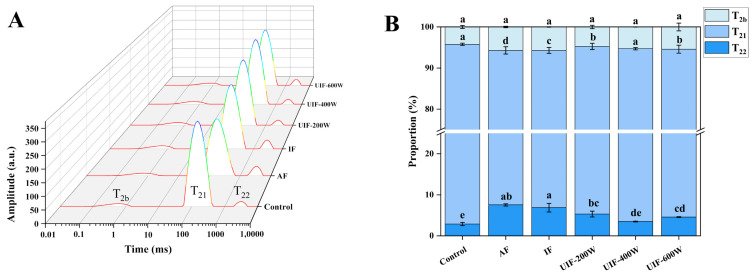
The impacts exerted by diverse freezing regimens on the relaxation times of T_2b_, T_21_ and T_22_ (**A**) and the proportions of water types (**B**) in MP gel. Significant differences among samples (*p* < 0.05) are denoted by different alphabetical notations.

Freezing damage will alter the native structure of MP, which further impairs the gel-forming ability and water retention capacity of thawed MP. Therefore, we characterized the moisture distribution of MP gels to evaluate the functional deterioration of protein induced by different freezing treatments. LF-NMR has become a common method for characterizing water molecular mobility and spatial distribution within protein gels non-destructively [58]. As shown in Figure 6A, all gels exhibited three peaks within the range of 0.1–10,000 ms, each representing a distinct water state: T_2b_ (0.1–10 ms) represents bound water, T_21_ (50–700 ms) immobilized water, and T_22_ (1000–5000 ms) free water [59]. Since T_2b_ accounts for a small proportion and is tightly bound to the protein surface in a stable state—less susceptible to external factors—it has little direct impact on WHC. In contrast, T_21_ constitutes the largest fraction of water in the gel, while T_22_ is highly mobile; both significantly influence water retention. Therefore, the relaxation times and peak area ratios of these two water populations served to characterize aqueous distribution within MP gels.

Shorter relaxation times indicate stronger binding and lower mobility of water, whereas longer times reflect higher mobility [60]. As can be seen from the figure, compared to the AF, the UIF-treated MP gels generally exhibited shorter relaxation times and a noticeable shift in the main peak position, indicating reduced water mobility and improved gel WHC. This is primarily because the fine ice crystals generated by UIF reduce mechanical damage to the myofibrillar structures, thus leading to less denaturation and more intact protein structure after extraction upon thawing. These structurally more complete proteins subsequently form a more uniform and dense 3D network during the heating and gelation process, which more effectively traps water and reduces its mobility [61]. As seen in Figure 6B, with the application of ultrasound, the peak area corresponding to free water decreased, while that of immobilized water (T_21_) gradually increased. The best result was achieved at UIF-400 W, most closely resembling the control (*p* > 0.05). This indicated that suitable ultrasonic power during freezing promotes the shift of free water to immobilized water within proteins, thereby enhancing the WHC in beef samples. In line with this, Zhang et al. [62] reported that ultrasound transformed more free water to immobilized water within surimi gels, leading to improved water retention in the sample.

### 3.9. Microstructure of MP Gel

#### 3.9.1. CLSM

**Figure 7:**
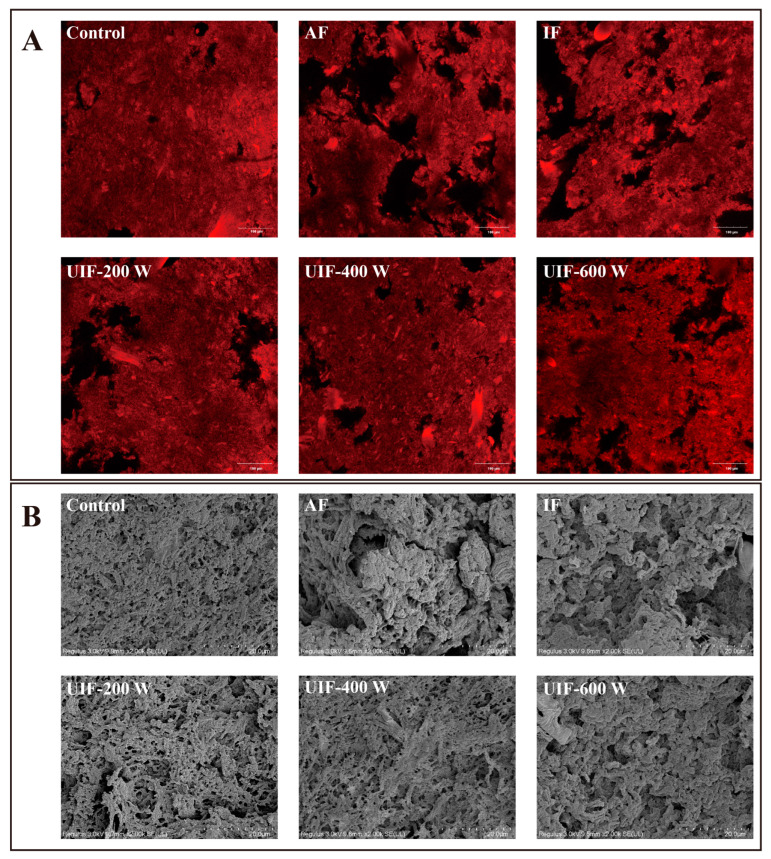
The microstructure of MP gels under different freezing treatments. CLSM (**A**), magnification: 200×; SEM (**B**), magnification: 2000×.

Figure 7A shows the microstructure of MP gels under different freezing treatments observed via CLSM, where the deep red regions represent dye-labeled protein phases, and the black regions correspond to protein-free pore areas. For the control, the continuous and uniform red phase indicates a dense, continuous gel network with minimal pores, which reflects the absence of freezing-induced damage. In contrast, the discontinuous red phase and disordered, large black pores in the AF indicate a discontinuous, loose, and porous protein gel structure; this is because large ice crystals formed during conventional freezing cause severe mechanical damage to the sample, leading to uneven protein aggregation and thus negative impacts on the gel structure, which aligns with the poor WHC of this group [61].

For the IF, the continuity of the protein network is slightly improved, but there are still many scattered medium-to-large pores, and the connectivity of the protein phase is limited, suggesting that conventional immersion freezing provides relatively weak protection for the gel structure. For the UIF-200 W and UIF-600 W, due to insufficient and excessive ultrasonic power, respectively, although the pore size is smaller than that in the AF, the network compactness remains inadequate, the protein phase distribution is poorly uniform, and a stable continuous structure cannot be formed. However, the protein gel in the UIF-400 W exhibits a continuous and dense network structure, with significantly fewer and smaller black pores; this is a direct manifestation of the ultrasonic cavitation effect refining ice crystals and reducing muscle fiber damage. The tight protein network not only provides more binding sites for water molecules but also corresponds to the superior WHC and gel elasticity of this group [63].

The above MP gel results can be well correlated with the quality attributes observed for the beef samples themselves. Specifically, UIF-400 W treatment not only resulted in a more uniform and dense MP gel network with improved water retention (as reflected by the increased T_21_ proportion and decreased T_22_ proportion), but also significantly enhanced the water-holding capacity of the intact beef, as evidenced by the reduced thawing and centrifugal losses (Section 3.1). This consistency suggests that the preservation of MP gelation ability directly contributes to improved moisture retention in the meat matrix. Furthermore, the superior G′ and the denser and more uniform microstructure of the MP gel from the UIF-400 W group corresponded well with the lower shear force values of the beef from the same treatment (Section 3.2), indicating that the integrity of MP gel network formation underpins the textural properties of cooked meat. Collectively, these correlations demonstrate that the macroscopic quality improvement of UIF-400 W-treated beef is mechanistically grounded in the protection of MP structural integrity and functionality during freezing.

#### 3.9.2. SEM

Figure 7B shows the SEM images, which further confirm the observations from CLSM at a more microscale. The MP gel of the control exhibits a fine, continuous 3D network with small, uniformly distributed pores, which aligns with the dense structure result from CLSM. All freezing-treated groups have reduced structural integrity: the networks of the AF and IF are rough and porous, with numerous large pores and cracks. While the network of the UIF-200 W is finer than those of the AF and IF, it still contains many scattered pores, indicating insufficient smoothness. The network of the UIF-400 W resembles the fine, continuous morphology of the control, which further proves that ultrasound-assisted freezing at an appropriate power effectively mitigates structural damage caused by ice crystal injury [40]. The network of the UIF-600 W appears rough with enlarged pore size; this may be attributed to the intense cavitation effect and microjets generated by excessive ultrasonic power, which damage beef muscle fibers and thus induce protein denaturation and structural disruption. Consistent with the above results, Zhang et al. [21] reported that excessive ultrasonic power leads to serious structural destruction of chicken breast myofibers.

### 3.10. Schematic Comparison of the Effects of AF and UIF

**Figure 8:**
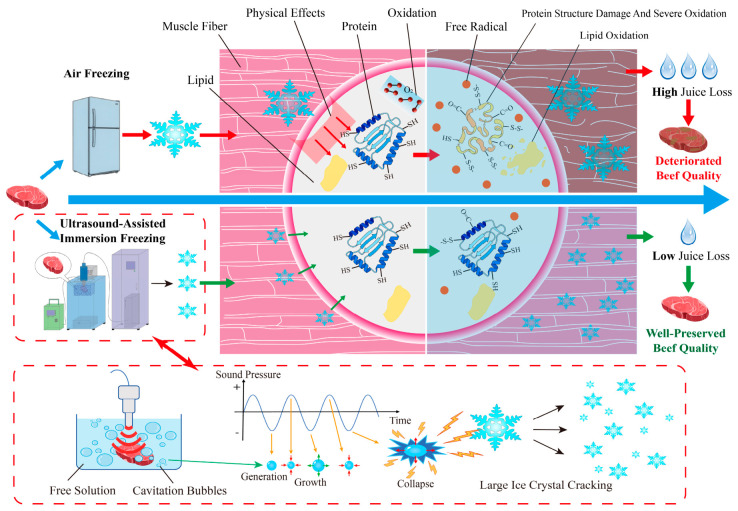
Schematic diagram of the mechanisms of AF and UIF on beef.

To visually illustrate the molecular mechanisms by which different freezing methods affect beef quality, a schematic diagram was prepared as shown in Figure 8. During the AF process, the slow freezing rate leads to the continuous growth of large ice crystals in the intercellular spaces, causing severe mechanical damage to muscle fibers and prolonging the window of oxidative stress, thereby exacerbating the oxidation and structural damage of proteins and lipids, ultimately resulting in deteriorated beef quality. In contrast, UIF generates cavitation bubbles in the freezing medium, where the bubbles expand during the negative-pressure phase and compress during the positive-pressure phase of the sound wave. After several cycles, the bubbles grow to a critical size and undergo violent collapse. The violent microjets produced during bubble collapse shatter large ice crystals into tiny homogeneous fragments. This effect greatly mitigates structural injury to muscle tissue, maintains the structural and functional integrity of proteins and lipids, and ultimately improves the quality of frozen beef.

## 4. Conclusions

This study systematically compared the effects of AF, IF, and UIF at different power levels on beef quality, oxidative properties, and myofibrillar protein functionality. The results demonstrated that UIF improved beef quality through dual physical and biochemical mechanisms. Physically, the microjets generated by ultrasonic cavitation broke large ice crystals into fine, uniform ones, effectively reducing mechanical damage to muscle fibers and cellular structures, thereby enhancing water-holding capacity and tenderness. Biochemically, the shortened freezing window and potential free radical scavenging effect synergistically inhibited oxidative reactions, delaying protein and lipid oxidation. Among all treatments, UIF-400 W exhibited the best performance: its carbonyl, sulfhydryl, and TBARS values showed no significant differences from the fresh control group (*p* > 0.05), and its macroscopic quality attributes such as water-holding capacity, tenderness, and color were also closest to those of the control group. Moreover, UIF-400 W effectively preserved the structural integrity and functional properties of myofibrillar protein.

In contrast, AF resulted in large ice crystals due to its slow freezing rate, exacerbating oxidative deterioration. UIF-200 W failed to induce effective cavitation due to insufficient power, while UIF-600 W caused excessive cavitation that damaged tissue and promoted oxidation. In conclusion, 400 W was identified as the optimal power for UIF treatment of beef. This technology effectively mitigates freezing-induced damage and preserves beef quality, providing a theoretical basis and practical strategy for producing high-quality frozen products in the meat industry.

Despite these promising results, the present study remains at the laboratory scale. We recommend the popularization and application of low-to-medium power ultrasound-assisted immersion freezing in small and medium-sized meat processing plants and household rapid freezers to mitigate quality loss of beef during frozen storage. Future research should evaluate longer frozen storage periods (e.g., 1 week, 1 month, and 1 year) to better simulate commercial storage conditions and to determine whether the protective effects of UIF-400 W are sustained over extended storage. Further exploration of the synergistic effects of UIF combined with other novel auxiliary freezing technologies, such as magnetic field-assisted freezing, is also warranted. In addition, integrating microbiological detection with proteomic and metabolomic analyses will help uncover the underlying molecular mechanisms governing quality and safety variations in frozen beef at a more profound level.

## Figures and Tables

**Table 1:** The thawing and centrifugation losses of beef under different freezing treatments.

Group	Thawing Loss (%)	Centrifugation Loss (%)
Control	— —	9.95 ± 0.20 ^e^
AF	6.58 ± 0.25 ^a^	16.87 ± 0.47 ^a^
IF	5.28 ± 0.18 ^b^	15.86 ± 0.46 ^b^
UIF-200 W	3.89 ± 0.16 ^c^	12.98 ± 0.37 ^c^
UIF-400 W	2.83 ± 0.07 ^d^	11.75 ± 0.21 ^d^
UIF-600 W	4.21 ± 0.25 ^c^	16.68 ± 0.67 ^a^

Note: Data are expressed as the means ± standard deviation. Significant differences among samples (*p* < 0.05) are denoted by different alphabetical notations.

**Table 2:** The impacts exerted by diverse freezing regimens on protein oxidation in beef.

Group	Total Sulfhydryl Content (nmol/mg·prot)	Carbonyl Content (nmol/mg·prot)	Ca^2+^-ATPase Activity (U/g)
Control	59.67 ± 0.31 ^a^	1.15 ± 0.03 ^e^	33.46 ± 1.07 ^a^
AF	43.41 ± 0.25 ^e^	1.77 ± 0.03 ^a^	21.68 ± 1.09 ^d^
IF	43.76 ± 0.41 ^e^	1.61 ± 0.03 ^b^	23.32 ± 0.66 ^c^
UIF-200 W	50.28 ± 0.31 ^c^	1.32 ± 0.05 ^d^	26.66 ± 0.23 ^b^
UIF-400 W	56.38 ± 0.09 ^b^	1.16 ± 0.03 ^e^	32.35 ± 0.60 ^a^
UIF-600 W	48.38 ± 0.67 ^d^	1.50 ± 0.04 ^c^	25.61 ± 0.54 ^b^

Note: Data are expressed as the means ± standard deviation. Significant differences among samples (*p* < 0.05) are denoted by different alphabetical notations.

## Data Availability

The original contributions presented in this study are included in the article. Further inquiries can be directed to the corresponding author.
